# Effects of metal cation substitution on hexavalent chromium reduction by green rust

**DOI:** 10.1186/s12932-020-00066-8

**Published:** 2020-02-14

**Authors:** Andrew N. Thomas, Elisabeth Eiche, Jörg Göttlicher, Ralph Steininger, Liane G. Benning, Helen M. Freeman, Dominique J. Tobler, Marco Mangayayam, Knud Dideriksen, Thomas Neumann

**Affiliations:** 1 Institute of Applied Geosciences, Karlsruhe Institute of Technology, 76137 Karlsruhe, Germany; 2 Institute of Synchrotron Radiation, Karlsruhe Institute of Technology, 76344 Eggenstein-Leopoldshafen, Germany; 3 GFZ German Research Center for Geosciences, Telegrafenberg, 14473 Potsdam, Germany; 4 Department of Earth Sciences, Free University of Berlin, 12249 Berlin, Germany; 5 Nano-Science Center, Department of Chemistry, University of Copenhagen, 2100 Copenhagen, Denmark; 6 Department of Applied Geosciences, Technical University of Berlin, 10587 Berlin, Germany; 7 Present Address: School of Chemical and Processing Engineering, University of Leeds, Leeds, LS29JT UK

**Keywords:** Chromium, Green rust, X-ray absorption spectroscopy, Remediation

## Abstract

Chromium contamination is a serious environmental issue in areas affected by leather tanning and metal plating, and green rust sulfate has been tested extensively as a potential material for in situ chemical reduction of hexavalent chromium in groundwater. Reported products and mechanisms for the reaction have varied, most likely because of green rust’s layered structure, as reduction at outer and interlayer surfaces might produce different reaction products with variable stabilities. Based on studies of Cr(III) oxidation by biogenic Mn (IV) oxides, Cr mobility in oxic soils is controlled by the solubility of the Cr(III)-bearing phase. Therefore, careful engineering of green rust properties, i.e., crystal/particle size, morphology, structure, and electron availability, is essential for its optimization as a remediation reagent. In the present study, pure green rust sulfate and green rust sulfate with Al, Mg and Zn substitutions were synthesized and reacted with identical chromate (CrO_4_^2−^) solutions. The reaction products were characterized by X-ray diffraction, pair distribution function analysis, X-ray absorption spectroscopy and transmission electron microscopy and treated with synthetic δ-MnO_2_ to assess how easily Cr(III) in the products could be oxidized. It was found that Mg substitution had the most beneficial effect on Cr lability in the product. Less than 2.5% of the Cr(III) present in the reacted Mg-GR was reoxidized by δ-MnO_2_ within 14 days, and the particle structure and Cr speciation observed during X-ray scattering and absorption analyses of this product suggested that Cr(VI) was reduced in its interlayer. Reduction in the interlayer lead to the linkage of newly-formed Cr(III) to hydroxyl groups in the adjacent octahedral layers, which resulted in increased structural coherency between these layers, distinctive rim domains, sequestration of Cr(III) in insoluble Fe oxide bonding environments resistant to reoxidation and partial transformation to Cr(III)-substituted feroxyhyte. Based on the results of this study of hexavalent chromium reduction by green rust sulfate and other studies, further improvements can also be made to this remediation technique by reacting chromate with a large excess of green rust sulfate, which provides excess Fe(II) that can catalyze transformation to more crystalline iron oxides, and synthesis of the reactant under alkaline conditions, which has been shown to favor chromium reduction in the interlayer of Fe(II)-bearing phyllosilicates.

## Introduction

Chromium is a common groundwater contaminant suitable for remediation by in situ chemical reduction [1, 2]. Geogenic chromium is associated with surficial ultramafic outcroppings, while anthropogenic chromium contamination typically results from chromium mining, metal plating facilities, tanneries and wood and paper treatment plants [3]. Once chromium enters soil and groundwater, its solubility and toxicity depend on its chemical speciation. In its trivalent form, chromium is insoluble and non-toxic, and is even an essential trace metal for sugar metabolism [4]. However, in its hexavalent oxidation state, chromium takes the form of chromate (CrO_4_^2−^), a highly soluble, toxic and carcinogenic compound. Redox transformations between the two forms occur in response to changing redox conditions; these transformations are often mediated by other metal biogeochemical cycles. Cr(III) oxidation to Cr(VI) is primarily mediated by biogenic Mn(IV) oxides [5–7], and the synthetic counterpart δ-MnO_2_ has been used to assess the lability of synthetic Cr(III)-bearing phases [8, 9]. However, no published study to date has used this method to assess the stability of Cr(III) carrier phases generated by a lab-scale in situ chemical reduction study.

Because the trivalent form of Cr is less soluble and toxic than its hexavalent form, chemical reduction of Cr is a potential remediation strategy referred to as in situ chemical reduction (ISCR) when used for remediation purposes. However, to work effectively, it is advantageous that any applied reduction method produces an insoluble Cr(III)-bearing product that is resistant to oxidation. At earth surface conditions, Cr(III) typically precipitates as a poorly-crystalline hydroxide [10, 11], which is vulnerable to dissolution and subsequent re-oxidation. On the other hand, coprecipitation of Cr(III) with Fe oxides results in a Cr(III) carrier phase that is more insoluble and resistant to oxidation. Therefore, any applied ISCR method should attempt to produce a Cr(III)-substituted Fe(III) oxyhydroxide product.

Green rust (GR) is a Fe (II)–Fe (III) layered double hydroxide (LDH) and has been shown to effectively reduce various contaminants causing their immobilisation, including chromium, yet in many cases the actual reduction mechanisms are still unclear. Green rust is composed of brucite-like Fe(OH)_2_ sheets in which a portion of the Fe^2+^ has been replaced by Fe^3+^, giving the sheets a positive charge. This positive charge is balanced by interlayer anions, where cations such as Na^+^ are also present [12]. There are two types of green rust, distinguished by their interlayer spacings and associated anions. Green rust 1 has a narrow interlayer spacing of ~ 8 Å occupied by chloride or carbonate, while green rust 2 has a broad interlayer spacing (~ 11 Å) typically occupied by sulfate, which allows exchange of tetrahedral oxyanions and subsequently reduction and sequestration of these substances in the reaction product’s interlayer [13–15]. Therefore, it is a promising reagent for exchange and/or reduction of selected groundwater contaminants such as As [16, 17], NO_3_^−^ [18, 19], U (VI) [15, 20], Se (VI) [21, 22], Np [23] and Cr(VI) [13, 14, 24–28].

The most commonly-identified product of chromate reduction by green rust is a poorly-crystalline Cr(III)–Fe (III) oxyhydroxide [13, 24, 25] or a Cr(III)–Fe (III) oxyhydroxycarbonate when green rust carbonate is used [26, 27]. However, Cr(III)-bearing goethite has also been observed to form [14] at the green rust particle rims when Cr concentrations are high and an excess of green rust is added to a batch reaction. Bond and Fendorf [13] and Skovbjerg et al. [14] concluded that these products formed due to exchange of chromate for interlayer sulfate followed by reduction. More recently, our previous study [28] reacted green rust with a series of initial chromium concentrations typical of contaminant plumes and determined that the speciation of chromium in the reaction product is correlated to the initial concentration. Although more goethite was found in the reaction products formed at higher initial concentrations, Cr(III) hydroxide, presumably located on the oxidized green rust particle surfaces, was the primary Cr(III) carrier phase produced. A similar side product was also identified by Legrand et al. [27]. The variable Cr(III) carrier phases identified under varying reaction conditions suggest that several reaction mechanisms are possible: reduction at the particle surface coupled to electron donation from the particle’s interior is expected to produce Cr(III) hydroxide, while Cr(III)-bearing Fe(III) oxyhydroxides can form when Cr(VI) is reduced in the interlayer following exchange of chromate for sulfate.

Like magnetite, green rust is a low-bandwidth semiconductor, and electron transfer from structural Fe(II) in the particles’ interior to the surface is possible via a polaron hopping mechanism [29]. A polaron is a quasiparticle consisting of an electron (hole) and the associated distortions in the surrounding lattice [30]. According to the polaron hopping model, which matches empirical observations [31, 32] of electron conductivity in metal oxides and other polaronic insulators, electron conduction can only take place via Fe(II)–Fe(III) charge transfer steps, each of which depends on superexchange coupling induced by intermediate cation-centered octahedra [29], as this transition would otherwise be spin-forbidden [33]. Incorporation of divalent and trivalent cations that have no net spin and only a single available oxidation state (e.g. Al^3+^, Mg^2+^ and Zn^2+^) may prevent or slow regeneration of Fe(II) at the particle surface, as these cations canot accept or donate electrons as part of a transfer chain [34] and may interfere with the superexchange coupling [35] that drives the rapid electron transfer modelled by Wander et al. [29]. In this case, Cr(VI) may only be able to access Fe(II) in the green rust crystal interior by exchanging for interlayer sulfate, which would lead to Cr(III) incorporation into a Fe(III) oxide product, although passivation of the particle may be an issue, as formation of an interlayer precipitate may hinder access to interior Fe(II). Since reduction at the surface depends on conduction of interior electrons to the surface, changes in the green rust particle’s electrical conductivity can lead to changes in the dominant reaction mechanism. Despite this, few studies have measured the Cr reactivity of green rusts with cation impurities incorporated into the octahedral layer. Ruby et al. [36] investigated the structure and formation of Al-substituted green rust sulfate, while recent studies ofits reactivity with hexavalent chromium found that it reduced Cr(VI) more quickly than unsubstituted green rust, suggesting that cation-substituted green rusts may be more effective in situ chemical reduction reagents than the pure form. Green rust sulfates with isomorphic substitutions of Mg^2+^ [37] and Zn^2+^ [38] have also been synthesized, but no published study has investigated their reactivity.

Previous investigations of chromate reduction by green rust have returned inconsistent results, possibly due to variations in synthesis techniques and reaction conditions across multiple studies. In the present study, pure sulfate GR and sulfate GR with isomorphic substitution of Al, Mg, and Zn were synthesized and reacted with Cr(VI). The lability of Cr(III) in the reaction products was then determined by measuring the release of Cr(VI) after treatment with synthetic δ-MnO_2_, the synthetic counterpart of biogenic Mn oxide which has been used to assess Cr lability in previous studies [7–9]. The structure and Cr speciation of these products were also determined using transmission electron microscopy (TEM), X-ray absorption spectroscopy (XAS), X-ray diffraction (XRD) and pair distribution function (PDF) analysis.

## Methods/experimental

### Green rust and feroxyhyte synthesis and characterization

All green rusts (green rust sulfate, Al-GR, Mg-GR, Zn-GR) were synthesized using the method from Géhin et al. [39] with metal sulfate salt reagents added to N_2_-purged Milli-Q water. The total metal concentration in all synthesis batches was 0.1 mol kg^−1^, with divalent to trivalent cation ratios of 3:1. To synthesize the substituted green rusts, the synthesis solutions prior to titration by NaOH were prepared by replacing 10% of the Fe^2+^ or Fe^3+^ by the desired cation (see Table 1). After synthesis, the green rusts were aged in solution for 48 h. The composition of the solid phase was then calculated by subtracting the values measured using ICP-OES, and aqueous Fe^2+^ concentrations were measured using the ferrozine method [40] after centrifuging the green rust suspensions and filtering the supernatant using the aforementioned 0.2 µm syinge filters. Feroxyhyte (δ-FeOOH) was synthesized using a method utilizing rapid Fe^2+^_(aq)_ oxidation by H_2_O_2_ [41] and used as a characterization standard for the reacted green rust samples.

**Table 1:** Summary of green rust chemical compositions and associated Fe^2+^ concentrations

Reactant	Expected chemical composition^a^	[Fe^2+^]_(aq)_ (mmol L^−1^)^b^
GR-SO_4_	$Fe_{4}^{II} Fe_{2}^{III} \left( {OH} \right)_{12} SO_{4} \cdot2H_{2} O$	25
Al-GR	$Fe_{4}^{II} Fe_{1.9}^{III} Al_{0.1} \left( {OH} \right)_{12} SO_{4} \cdot2H_{2} O$	27
Mg-GR	$Fe_{3.8}^{II} Mg_{0.2} Fe_{2}^{III} \left( {OH} \right)_{12} SO_{4} \cdot2H_{2} O$	23
Zn-GR	$Fe_{3.8}^{II} Zn_{0.2} Fe_{2}^{III} \left( {OH} \right)_{12} SO_{4} \cdot2H_{2} O$	23

^a^Based on ratio of cations in solution prior to titration

^b^Expected [Fe^2+^]_(aq)_ is 20–25 mmol L^−1^

### δ-MnO_2_ synthesis

Vernalite (δ-MnO_2_), which resembles natural biogenic Mn (VI) oxides [42], was synthesized using the “redox” method of Villalobos et al. [43]. MnCl_2_ was added slowly to a KMnO_4_ solution while maintaining a pH of 7 using NaOH. The product was first rinsed several times with 1 M NaCl to remove the remaining Mn^2+^, then with Milli-Q water before further purification using dialysis. Vernalite was kept in suspension by sonication and adjusted to pH 7.5 before use in re-oxidation batch reactors.

### Batch reactions

Three replicate batch reactors were set-up for each synthesized green rust type: one for solid phase characterization and two to measure Cr(VI) reduction and Cr(III) re-oxidation by δ-MnO_2_. All batch reactions were performed in an anaerobic chamber with an Ar atmosphere. In each reactor, an aliquot of green rust suspension with about 0.2 mmol of Fe (II) was added to a 100 mL 0.67 mmol kg^−1^ K_2_CrO_4_ solution in an acid-washed borosilicate beaker ([Fe(II)]/[Cr(VI)] ≤ 3, slight excess of Cr(VI) to ensure complete oxidation and prevent the Fe^2+^-catalyzed transformation of reaction products), with the pH of all solutions adjusted to 7. The batch reactions were not shielded from the light as this would have prevented sample removal, and the reaction temperature and pH were not controlled to allow direct comparison to similar studies that followed the same procedure [14, 28]. The first reaction was terminated after 7 days by filtration (0.2 μm, Whatman nylon membrane filter), and solid samples were removed for further characterization. Aging for 7 days allowed incipient transformation of the initial metastable intermediate [14, 28]. In the second and third reactors, suspension aliquots were periodically removed and filtered during the first hour of the reaction to monitor the removal of Cr(VI) by GR reduction; after 7 days, colloidal, synthetic δ-MnO_2_ was added to the remaining suspension. Samples removed prior to and 1 and 2 weeks after δ-MnO_2_ addition were treated with 10 mM Na_2_HPO_4_ for 24 h to desorb chromate from mineral surfaces, followed by filtration. [Cr(VI)] in all samples was measured using the 1,5-diphenylcarbazide method (US Environmental Protection Agency (EPA) method 7196A) with a Perkin-Elmer Lambda 2S UV-Vis Spectrophotometer calibrated using a four-point calibration curve. In addition, measurement of [Fe^2+^_(aq)_] by the ferrozine method was attempted, but the results are not shown here because [Fe^2+^_(aq)_] decreased to below the limit of detection within ten seconds.

### X-ray diffraction (XRD) and pair distribution function (PDF) analyses

Benchtop XRD measurements were performed using a Bruker D8 Diffractometer. Unreacted green rust samples were removed from suspension by filtration, treated with glycerol to prevent oxidation, and transferred as a paste to a standard Si powder specimen holder. X-rays were emitted from a Cu-Kα source (λ = 1.5418 Å), and data were collected at 2θ values between 2 and 82° with a step size of 0.02° and an average counting time of 1 s per step. Background diffraction patterns were collected by measuring an empty sample holder, and the XRD-BS software was used to remove the background from the sample data.

Synchrotron X-ray scattering measurements of reacted samples were performed at beamline 11-ID-B at the Advanced Photon Source (APS) at Argonne National Laboratory, using an X-ray energy of 58.66 keV (λ = 0.2113 Å). Samples were ground and transferred into glass capillaries sealed with paraffin, then measured at a distance of ~ 18 cm (PDF) and 100 cm (XRD) using a 40 cm × 40 cm amorphous Si 2D detector. An empty glass capillary and a CeO_2_ standard were also measured for background subtraction and calibration of the Laue patterns, respectively. The collected patterns were converted to 1D data using the Fit2D software after calibrating the geometry of the setup using the CeO_2_ standard. For high resolution XRD, the I(Q) data collected at 100 cm was treated with the software GSAS-II [44] to perform background subtraction, and to convert the incident beam energy to Cu-Kα (λ = 1.5406 Å) for comparison with lab based XRD. Full width half maximum (FWHM) values for the green rust {213} reflection were determined using the peak fitting extension in OriginPro 2018. PDF patterns were extracted from the data collected at 18 cm using the software PDFGetX3 [45], including background subtraction and corrections for incoherent scattering and non-linear detector efficiency as well as normalization to the sample’s average atomic scattering cross-section [46]. The composition of the sample was set at Fe_0.5_Cr_0.12_O_0.38_ due to the stoichiometry of the reaction. Fourier transformation of the reduced structure function Q[S(Q)^−1^] was performed using a maximum Q-value of 20 Å^−1^ to yield G (r), the reduced pair distribution function.

### X-ray absorption spectroscopic (XAS) analyses and data processing

Bulk XAS spectra of all reacted samples were collected at the SUL-X beamline at the ANKA synchrotron facility in Eggenstein-Leopoldshafen, Germany, which operates at 2.5 GeV. The incident beam was scanned through the Cr and Fe K-edges (set at 5989 eV and 7112 eV for metallic Cr and Fe, respectively), using a Si (111) crystal pair monochromator with a fixed-beam exit. Higher-order harmonics were removed from the incident beam using a grazing incidence mirror. Three replicates of each spectrum were measured in both transmission and fluorescence mode in a range of − 200 to 1000 eV relative to the absorption edge. Transmission spectra were obtained using three Oxford Instruments IC-type ionization chambers with Kapton windows, and fluorescence measurements were obtained using a Gresham 7-element Si (Li) detector. All spectra were calibrated with a Cr or Fe metal foil placed between ionization chambers 2 and 3.

Fe and Cr XAS data were processed and analyzed using the Demeter software package [47]. All analyses were performed on spectra obtained in transmission mode. After calibrating the spectral energies using reference metal foil spectra, a merged spectrum was produced by averaging the three replicate spectra in μ(E)-space. The merged spectra were then normalized using a first-order pre-edge function and a third-order post-edge spline function to model the background absorption.

For Cr X-ray absorption near-edge spectroscopy (XANES) linear combination fitting, the derivative of each μ(E) spectrum was fitted to two reference standards (Cr(III)-bearing ferrihydrite and Cr(III) hydroxide [48, 49]) in the XANES region (− 20 to + 30 eV). No further constraints were placed on the fit. Fe K-edge extended X-ray absorption fine-structure spectroscopy (EXAFS) fitting was also performed, and due to the similarity of the spectra to feroxyhyte, the procedure for fitting of a feroxyhyte spectrum outlined in Manceau and Drits [50] was followed. Where possible, the *k*^*3*^-weighted EXAFS spectra were Fourier transformed over a *k*-range of 4–14 Å^−1^; other ranges were used when the data at high *k*-values was too noisy or a Co K-edge was present in this region (oxGR). Shell-fitting was performed using the Artemis software. Theoretical Fe phase and amplitude functions were calculated from the crystal structure of hematite [51] with no further modifications. During fitting, S_0_^2^ was fixed at 0.9 and all σ^2^ values were fixed at 0.015, as the fitted value of this parameter tended to converge to 0.015 when the fits were performed. All fits were performed using three single-scattering paths: Fe–O (1.98 Å), edge-sharing Fe–Fe (3.01 Å), and corner-sharing Fe–Fe (3.4 Å). Other single- and multiple-scattering pathways were tested but ultimately excluded because they failed to improve the fit or produced unphysical parameter solutions. A more detailed outline of the Fe K-edge EXAFS fitting procedure is included in Additional file 1: Appendix 2.

### Electron microscopy

Scanning electron microscopy (SEM) images of unreacted green rusts were recorded on an FEI Quanta 3D FEG microscope. Samples were prepared inside an anaerobic chamber by filtering an aliquot of a green rust suspension through a 0.2 μm nylon filter, and the paste was transferred to an SEM sample holder. The samples were then immediately transferred to the microscope’s vacuum chamber to prevent oxidation. Images were obtained in high vacuum mode at an accelerating voltage of 20 kV using an Everhart-Thornly secondary electron detector.

Higher resolution images of green rust samples reacted with Cr(VI) were recorded using a TEM on samples prepared by transferring several drops of a sonicated suspension in ethanol to a 3 mm Cu-TEM grid coated with a holey amorphous carbon film. The TEM grids were then transferred to a FEI Tecnai TEM operated at 200 kV and equipped with a Gatan Tridiem imaging filter (GIF), a Fishione high-angle annular dark field detector, an energy dispersive X-ray (EDX) analyzer to measure chemical composition and a Gatan Orius SC200D 4 K pixel cooled CCD camera. Selected-area electron diffraction (SAED) patterns were collected using plates with an aperture of ca. 200–300 nm and developed in a Ditabis Imaging Plate Scanner. The d-spaces and FWHM values were calculated from manual measurements obtained using the ImageJ software. TEM images were processed and converted using Gatan DigitalMicrograph, while the raw EDX data was processed using EDX Quant.

## Results and discussion

### Characterization, reduction of Cr(VI) and reaction product stability

Based on the measurements shown in Table 1, all green rust suspensions have similar aqueous Fe^2+^ concentrations which are similar to the expected concentrations based on the 2:1 ratio of Fe(II) to Fe(III) in green rust sulfate. Mg- and Zn-GR are associated with slightly lower Fe^2+^ concentrations, however, most likely because 10% of the Fe(II) in these suspensions (compared to pure GR) is replaced by Mg or Zn. As aqueous Fe^2+^ can also reduce Cr(VI) [11, 48, 52], the inconsistent Fe^2+^ concentrations shown in Table 1 may result in differing Cr-bearing phases in the products of these reactions, as discussed later.

**Fig. 1:**
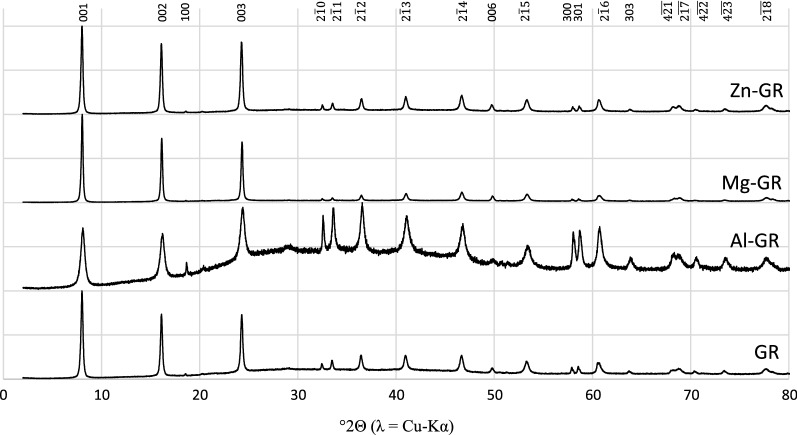
XRD patterns obtained after aging synthetic, unreacted green rusts for 24 h. Miller indices apply to all diffraction patterns where the selected reflection is present and are assigned based on the green rust sulfate structure from Simon et al. [53]

**Fig. 2:**
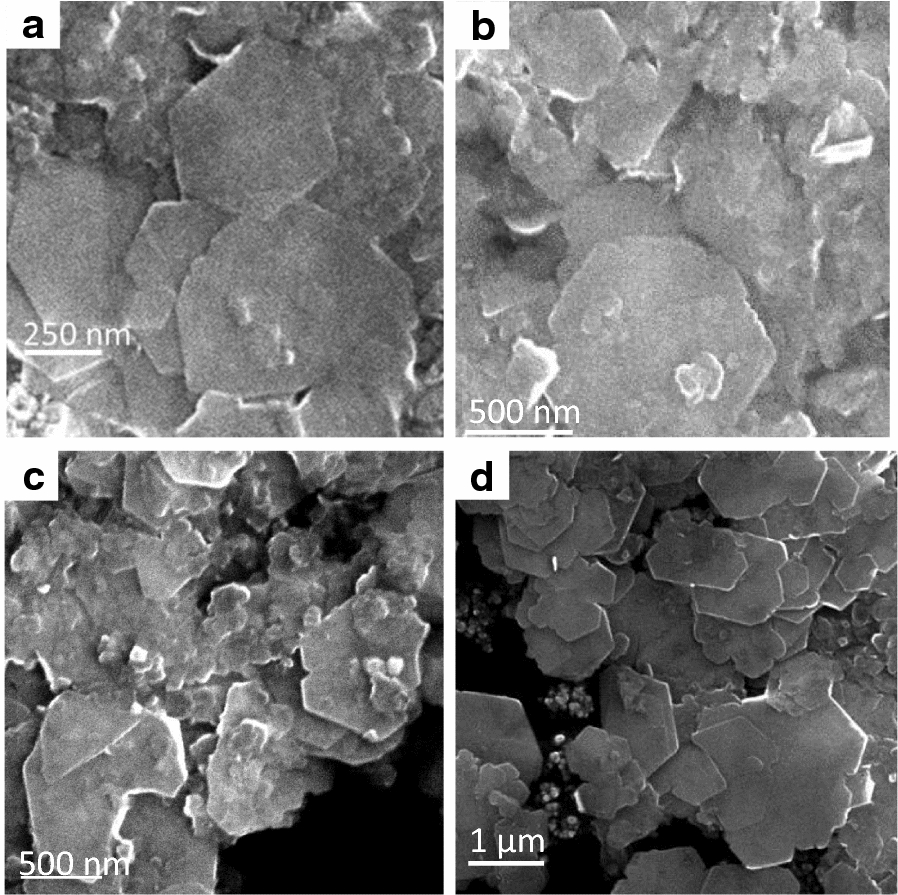
SEM micrographs of GR (**a**), Al-GR (**b**), Mg-GR (**c**) and Zn-GR (**d**)

Figure 1 shows the benchtop XRD patterns of the synthetic green rusts prior to reaction with hexavalent chromium. All patterns had the same green rust 2 peaks predicted by the crystal structure from Simon et al. [53], but there were differences in peak amplitude and shape. Al substitution for Fe(III) resulted in an 87% increase in the FWHM of the {213} peak at 41° 2θ relative to GR, suggesting that Al substitution either increases the structural disorder or decreases the crystallite size in the green rust as observed in previous studies [36]. Representative SEM images (Fig. 2) show that all green rusts have a characteristic hexagonal morphology, but the substituted green rust particle morphologies are more irregular than pure green rust. In addition, it is clear that the substituted green rusts have broader particle size distributions, possibly due to non-uniform incorporation of Al, Mg and Zn.

**Fig. 3:**
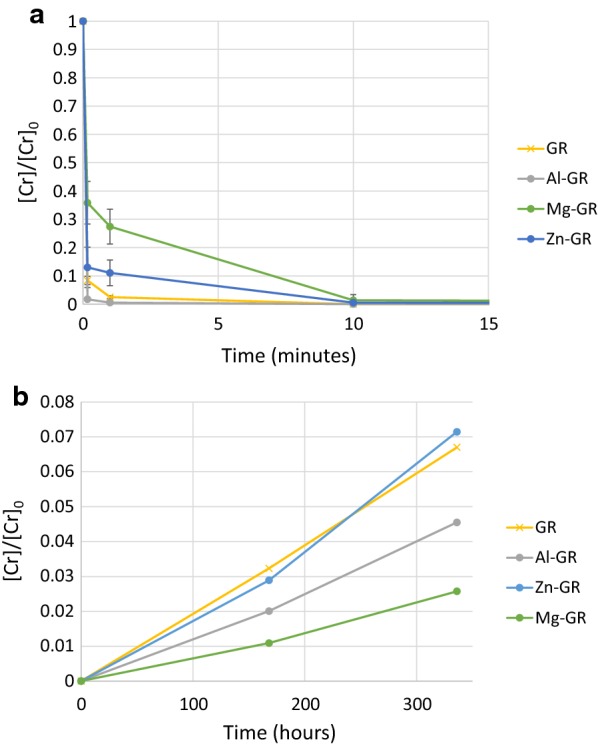
Kinetics of Cr (VI) reduction by the synthesized green rusts (**a**) and reoxidation of Cr (III) when exposed to synthetic, colloidal δ-MnO_2_ (**b**)

The removal of chromate by the various green rust sulfates is shown in Fig. 3a. In all cases, chromate concentrations are reduced to below the detection limit (0.04 mg kg^−1^, below the WHO-recommended limit of 0.05 mg kg^−1^) after 10 min of reaction time; however, Cr(VI) is removed from solution much more rapidly by pure green rust and Al-GR, while substitution by zinc and magnesium led to slower removal of Cr(VI) from solution. This may be due to differences in particle size (Fig. 2) and/or [Fe^2+^_(aq)_] concentration; several Mg-GR and Zn-GR particles with diameters near 700 nm are visible, possibly indicating that Mg-GR and Zn-GR have lower reactive surface areas, and Fe^2+^ reacts more rapidly with chromate than structural Fe(II) [52]. Our previous study [28] measured Cr(VI) reduction at different chromium concentrations, finding that green rust consistently reduces all chromium in solution, but the reaction rate decreases with the chromium concentration. It is unknown whether the differences in reaction rate between the green rusts tested in the present study are consistent at other chromium concentrations. Figure 3b shows the release of chromate, i.e. re-oxidation of Cr(III) to Cr(VI) by colloidal δ-MnO_2_. Approximately 7% of the reduced chromium in the pure green rust and Zn-bearing green rust reaction products were re-oxidized ([Cr] = 2.4 mg kg^−1^), compared to 2.5% ([Cr] = 0.9 mg kg^−1^) and 4.5% ([Cr] = 1.6 mg kg^−1^) of the Cr in the product of Mg- and Al-bearing green rust, respectively. These differences most likely result from differences in the particle structure and Cr speciation of the reaction byproducts, which are discussed below.

### Long-range order structure and particle morphology of reaction products

**Fig. 4:**
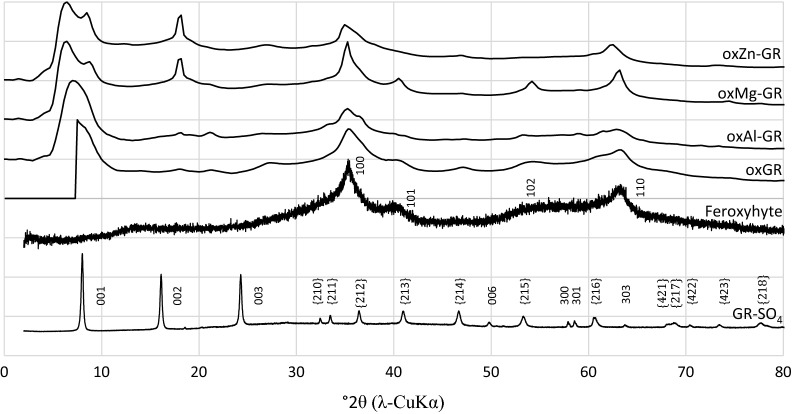
X-ray diffraction patterns of reacted samples and reference standards synthesized for comparison. X-axis recalculated to represent patterns in terms of 2θ (CuKα). Miller indices assigned based on unit cell structures of green rust sulfate (Simon et al. [53]) and feroxyhyte (Patrat et al. [54])

Synchrotron XRD patterns of solids formed after reacting the GRs with Cr for 7 days are shown in Fig. 4. For simplicity, all reaction products in this publication will be identified as oxGR (oxidized green rust sulfate) or ox*n*-GR (*n*=Al, Mg or Zn). Two broad reflections at 2.55 Å (2θ = 35.2°) and 1.46 Å (2θ = 63.7°) are the most notable features present in each pattern. These spacings were also observed in the oxidized green rust diffraction patterns from Skovbjerg et al. [14] and are characteristic of many Fe oxides with hexagonal symmetry such as ferrihydrite and feroxyhyte [54, 55] and most likely indicate a residual hexagonal symmetry remaining after oxidation and restructuring of the green rust. However, the broadness of these peaks suggests structural disorder in the [001] crystallographic direction. In the case of oxMg-GR, partial transformation to feroxyhyte is also evident, as the broad 2.55 Å and 1.46 Å reflections are sharper and several minor feroxyhyte reflections at ~ 40.5° (101) and 54.2° 2θ (102) are also visible in this XRD pattern. Every sample is also partially composed of a residual layered ferric green rust structure, as shown by a broad, diffuse reflection below 8° 2θ [14], which is much more prominent in the wet sample diffraction pattern (Additional file 1: Fig. S1) obtained using a Bragg-Bretano instrument. Therefore, the broadening of this peak is most likely due to drying of the sample prior to measurement, which can dehydrate the interlayer to a variable degree and cause stacking distance variability in the [001] direction. A reflection at about 10.4 Å (2θ = 8.5°) is also visible in the oxMg-GR and oxZn-GR patterns; this reflection is similar to the (001) reflection in green rust sulfate and suggests that the original, hydrated structure has been preserved to some extent. These reflections are also visible in Additional file 1: Figure S1, but the data is much noisier and many minor peaks are not visible. In addition, a minor reflection at 4.9 Å (2θ = 18.1°) is present in the oxMg-GR and oxZn-GR patterns, but the authors were unable to identify the source of this peak. This spacing does not correspond to any known iron oxide structure.

**Fig. 5:**
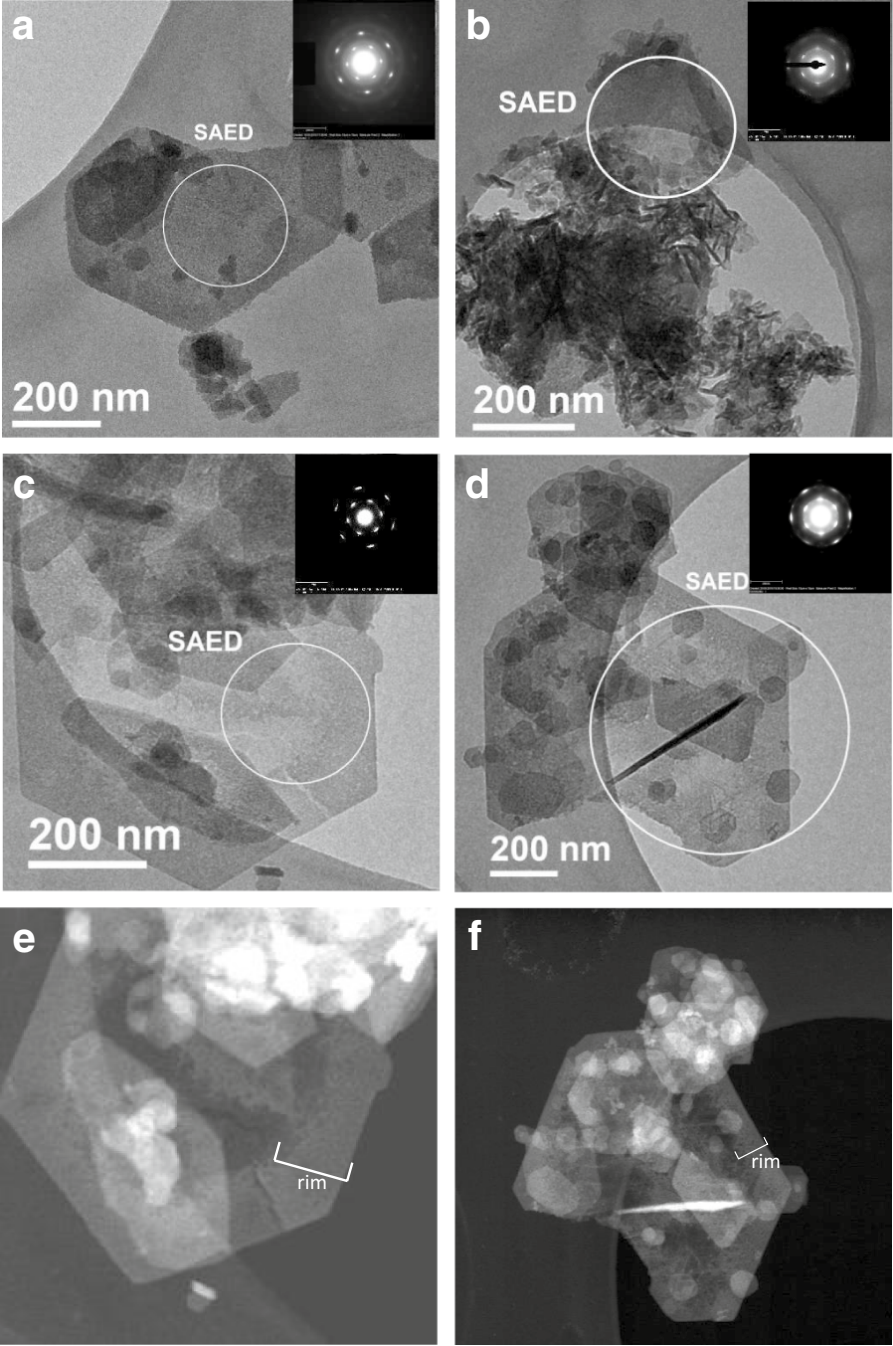
TEM images of oxGR (**a**), oxAl-GR (**b**), oxMg-GR (**c**) and oxZn-GR (**d**). STEM images of oxMg-GR (**e**) and oxZn-GR (**f**) are also shown. Areas where SAED was performed are indicated, and the SAED patterns are displayed in the insets. FWHM of diffraction spots (mm, at 2.53 Å/1.46 Å): **a** 0.65/1.26, **b** 0.71/1.51, **c** 0.55/0.67, **d** 0.72/1.07

Figure 5 shows TEM images of the various green rust samples after reaction with aqueous chromate. In all samples, the pseudo-hexagonal morphology of the reactant particles remains preserved after oxidation and the particle diameters are similar to those measured in Fig. 2, but the particle edges are more irregular, particularly in the case of oxAl-GR. This sample also has many irregular particular aggregates, but several pseudo-hexagonal particles are visible, particularly the particle from which the SAED pattern was obtained. Higher-contrast domains at the rims of some oxMg- and oxZn-GR hexagonal particles are visible, which are also associated with lower density in the interior domains of the same particle (more easily visible in the STEM (scanning transmission electron microscopy) images, Fig. 5e–f). Although particles with these rim-like domains are present in both oxMg-GR and oxZn-GR, they are more prevalent in oxMg-GR. Particles with similar morphologies were identified by Skovbjerg et al. [14], but the rim domains in the present study are more poorly-defined than the domains that formed at chromium levels high enough to oxidize 60% of the Fe(II) bound in green rust. This study concluded that these features formed due to Cr reduction by green rust from the rim inwards. Particles with other morphologies (i.e. rods and amorphous aggregates) are also visible.

In the SAED patterns collected from selected hexagonal particles, two hexagonal sets of reflections corresponding to d-spacings of ~ 2.5 and ~ 1.47 Å are also visible in all samples (see Fig. 5 insets) with variable sharpness. These patterns confirm the hexagonal symmetry of the product when observed from the [001] direction and indicate that there is some coherence between adjacent sheets along this axis. The oxidation product for which the smallest proportion of Cr was oxidized by δ-MnO_2_, oxMg-GR, had a SAED pattern with the lowest calculated peak FWHM values (mm, inner ring/outer ring = 0.55/0.67) when measuring in the direction of the center of the pattern, suggesting increased coherency between hydroxide sheets. The corresponding peaks (at 34° and 63.2° 2θ) are also sharpest in the oxMg-GR diffraction pattern (Fig. 4b).

Elemental concentration ratios measured by EDX and averaged over all measured hexagonal particles in each sample are shown in Table 2. Spectra taken from non-hexagonal particles were excluded from these calculations. These elemental ratios can determine whether certain elements are enriched or depleted in the various oxGR’s that formed during the reactions and also provide insights into the mechanisms that drive enrichment or depletion. Cr/Fe+Cr ratios are similar to the ratio (~ 0.18) predicted by the reaction’s stoichiometry (Eq. 1). The value of this ratio is not expected to vary for different topotactic reaction mechanisms, as three structural Fe (II) atoms are necessary to reduce one Cr(VI) atom in all cases. The Fe/S ratios, which can serve as proxies for exchange of interlayer sulfate, vary widely between samples, although many of these measurements have a large standard deviation. OxGR has a measured Fe/S ratio of 4.2, lower than the unreacted green rust ratio of 6.0 (Eq. 1) [12, 39], although the sample size of this measurement is not large enough to determine whether this difference is statistically significant.

**Table 2:** Elemental ratios determined by EDX. Values are averages calculated from measurements of all selected areas

Sample	[Cr]/([Cr+Fe])^a^	[Fe]/[S]
oxGR	0.19 (0.02) (n = 2)	4.21 (1.68) (n = 2)
oxAl-GR	0.142 (0.012) (n = 2)	13.90 (2.20) (n = 2)
oxMg-GR	0.158 (0.052) (n = 7)	31.19 (14.45) (n = 7)
oxZn-GR	0.179 (0.074) (n = 8)	13.72 (6.94) (n = 8)

Elemental ratios and uncertainties calculated from all EDX measurements taken from hexagonal particles

^a^Expected ratio (reaction stoichiometry) is ca. 0.18

1 $$0.75Fe_{4}^{II} Fe_{2}^{II} \left( {OH} \right)_{12} SO_{4} \cdot8H_{2} O + CrO_{4}^{2 - } + 0.5H^{ + } \mathop \to \limits^{{}} 5.5Fe_{0.818} {Cr}_{0.182} OOH + 0.75SO_{4}^{2 - } + 8H_{2} O$$

On the other hand, oxMg-GR has a significantly higher Fe/S ratio than oxZn-GR and the ratio in unreacted green rust sulfate (~ 6.0), and it is likely that sulfur has been depleted in this sample, possibly by chromate exchange for sulfate and its subsequent reduction in the interlayer.

### PDF and XAS characterization of short-range order structure of reaction products

**Fig. 6:**
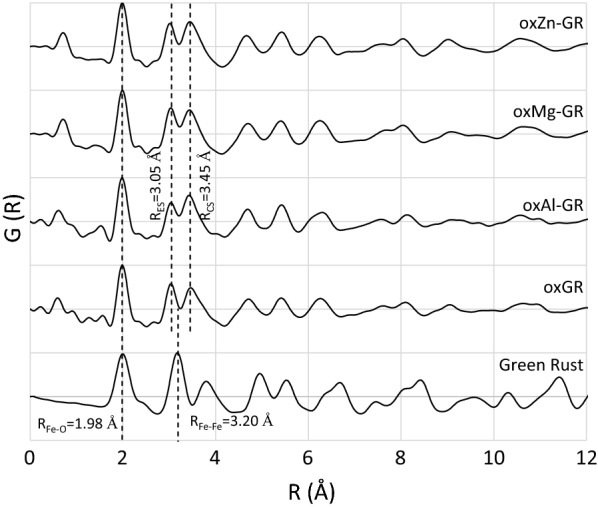
Calculated pair distribution functions [G(r)] for each measured reaction product, normalized to the intensity of the correlation at 1.98 Å. A Cr (III) hydroxide PDF pattern is not available, but has known correlations at 1.98 and 3.0 Å (Tang et al. [57]). Fe–O and Fe–Fe distances referenced in the text are labelled. R_ES_ and R_CS_ correspond to edge-sharing and corner-sharing Fe-Fe distances, respectively

The reduced pair distribution functions G(r) calculated for all reaction products produced in this study (Fig. 6) are nearly identical to those observed by Yin et al. [56], who characterized the oxidation products of 3:1 (i.e. [Fe(III)]/[Fe(II)] = 3) oxidized green rust chloride single sheets separated by dodecanoate intercalation (single-sheet iron oxide, SSI) and green rust sulfate oxidized by an excess of Cr(VI), respectively. These studies hypothesized that oxidation of the brucite-like layer caused dislocation of part of the Fe (III) into the interlayer, which manifests in the PDF as a splitting of the single green rust peak at ~ 3.20 Å [Fe(II)–Fe(II) and Fe(II)–Fe(III) edge sharing] into two peaks at approximately 3.04 Å [Fe(III)–Fe(III) edge-sharing] and 3.41 Å [Fe(III)–Fe(III) corner sharing], both of which can be seen in the reaction products in Fig. 6, although the peak positions differ slightly (3.05 and 3.45 Å). The XRD (Fig. 4) and SAED (Fig. 5) patterns indicate that there is some coherence between the stacked, oxidized layers, but the associated inter-sheet correlations most likely have amplitudes too low to be visible in the PDF patterns, as random Fe dislocations may remove atomic correlations while leaving the stacking in the [001] direction coherent enough to generate identifiable XRD and SAED reflections.

However, there are significant differences in peak intensity from the Yin et al. [56] PDFs at low R-values, particularly between 3.0 and 3.5 Å. The amplitudes of the peaks at 3.05 Å and 3.45 Å are very similar in the present study’s PDF patterns while the intensity at 3.41 Å in Yin et al. [56] is greater. Oxidation of 2:1 green rust sulfate (i.e. [Fe(II)]/[Fe(III)] = 2) produces a lower layer charge than oxidation of 3:1 green rust chloride, and therefore requires a smaller degree of internal rearrangement and deprotonation/hydroxylation to balance this layer charge. In addition, Tang et al. [57] found that PDF patterns of Cr(III) hydroxide have prominent pair correlations at 1.98 Å and ~ 3.0 Å, which may also contribute to the amplitudes of the peaks at 1.98 and 3.03 Å in Fig. 6, but do not have high enough amplitudes at higher R to contribute to the pattern in this region due to the small (> 10 Å) domain sizes.

**Fig. 7:**
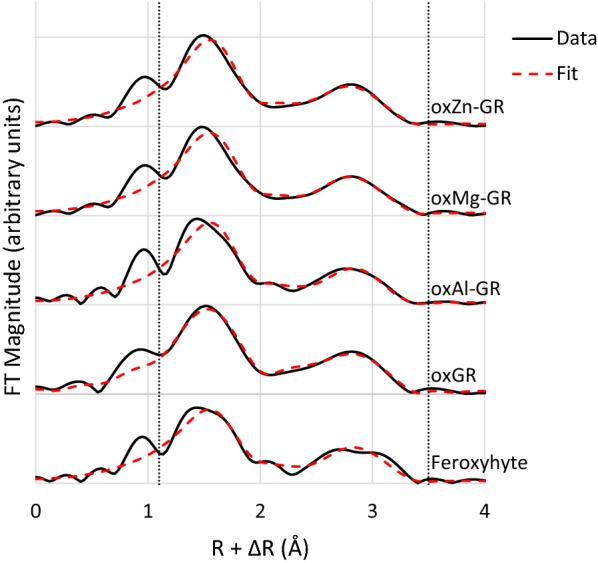
Fourier transformed Fe K-edge EXAFS spectra and fits after 7 days reaction time, as well as a feroxyhyte spectrum for comparison . Fit statistics and sample-specific fitting parameters outlined in Table 3. Fits performed over an R-range of 1.1–3.5 Å

Figure 7 shows the Fe EXAFS shell-by-shell fits of all reacted green rust products and a feroxyhyte reference standard for comparison, and the associated fit results are listed in Table 3. The short-range (< 4 Å) bonding environment of Fe in these samples is characterized by a mix of edge- and corner-sharing MeO_6_ octahedral linkages at distances of ~ 3.04 and 3.4 Å, which matches the structure predicted by PDF. The second- and third-shell coordination numbers have ratios similar to the apparent intensity ratios of the corresponding PDF peaks (Fig. 6), and the sums of these coordination numbers are all close to six. Because each Fe octahredron is also surrounded by six Fe in unreacted green rust sulfate, this supports the formation mechanism suggested by the PDF, as Fe dislocated into the interlayer is still bound to Fe remaining in the octahedral layer. The Fe EXAFS spectra also show that the Fe-bearing phases in all samples resemble feroxyhyte, particularly oxMg-GR, but there are significant differences. All samples have more edge-sharing Fe than feroxyhyte, particularly oxGR and oxZn-GR as well as oxMg-GR, even though feroxyhyte is identifiable in the latter sample by XRD. This may be due to features such as the brucite-like sheet inherited after the transformation, as this sheet, which is dominated by edge-sharing linkages, has fewer vacancies than a similar feroxyhyte structure [54, 58] if the transformation is topotactic. In addition, the two shells at 3.04 and 3.41 Å are distinct in the feroxyhyte spectrum but merged into a single shell in the sample spectra, suggesting that these samples have a significantly higher degree of structural disorder than synthetic feroxyhyte.

**Table 3:** EXAFS fitting parameters for all Fe K-edge EXAFS spectra

Sample	*k*-*range*	R-value	S_0_^2^	Shell: ΔE_0_ (eV)	*Fe*-*0* CN	R (Å)	σ^2^ (Å^−2^)	*Fe*–*Fe (edge*-*sharing)* CN	R (Å)	σ^2^ (Å^−2^)	*Fe*–*Fe (corner*-*sharing)* CN	R (Å)	σ^2^ (Å^−2^)
Feroxyhyte	4–14	0.014	0.9*	− 4.19	6*	1.98	0.013	2.80 ± 13%	3.03	0.015*	2.39 ± 20%	3.41	0.015*
oxGR	4–11	0.004	0.9*	− 4.63	6*	1.98*	0.011	3.88 ± 10%	3.05	0.015*	2.49 ± 20%	3.41	0.015*
oxAl-GR	4–14	0.011	0.9*	− 4.56	6*	1.98*	0.012	3.19 ± 12%	3.04	0.015*	2.59 ± 20%	3.38	0.015*
oxMg-GR	4–13	0.008	0.9*	− 4.11	6*	1.98*	0.012	3.18 ± 10%	3.02	0.015*	2.23 ± 20%	3.41	0.015*
oxZn-GR	4–13	0.006	0.9*	− 4.2	6*	1.98*	0.012	3.73 ± 10%	3.05	0.015*	2.26 ± 20%	3.41	0.015*

*Parameter was fixed during fitting

**Fig. 8:**
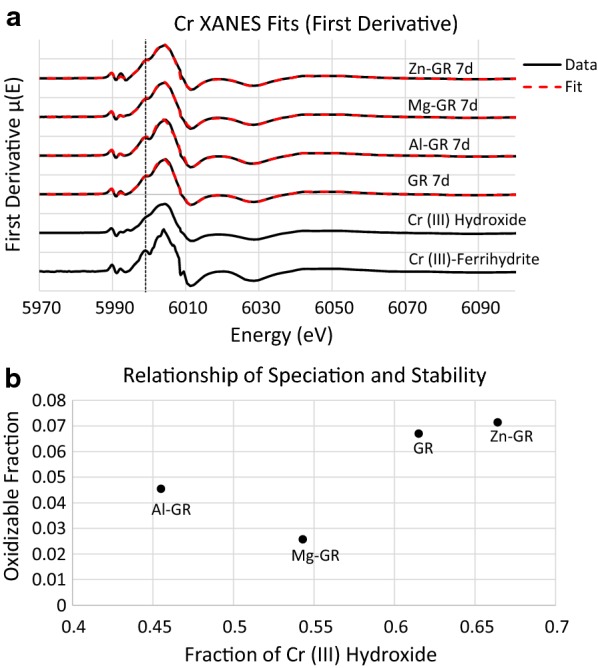
Cr K-edge XANES fits of the first derivative μ (E) for each measured sample (**a**). Fit performed between E-values of 5984 and 6034 eV. Dotted line at 5999 eV indicates location of pre-edge feature characteristic of Cr (III) hydroxide. **b** Compares the results of these fits to the 14-day oxidizable fractions shown in Fig. 3b

XANES linear combination fits were also performed on all Cr K-edge XAS spectra, using synthetic Cr(III) hydroxide and Cr(III)-bearing ferrihydrite as reference standards (Fig. 8a, fit results and statistics shown in Table 4). Cr(III)-bearing ferrihydrite, which is used here in the absence of other Cr(III)-bearing Fe oxyhydroxide reference standards, can be identified in the XANES spectra by characteristic pre-edge features at 5993 and 5999 eV visible in the derivative of the μ (E) spectrum [57]. EXAFS fits were not performed due to the difficulty of differentiating scattering by Fe and Cr; the distortion of the immediate (hydr)oxide bonding environment as Cr is incorporated into increasingly crystalline solids is more easily detectable using XANES fitting [57]. The proportion of Cr(III) hydroxide in each fit varies between 0.54 and 0.66. The proportions of each reference standard fit to the spectra have relatively high errors, but as the amount of Cr(III) hydroxide detected in each sample and the fitted values match the relative prominence of the characteristic Cr(III)-ferrihydrite features, errors in these values are unlikely to be as high as suggested by the fitting software. Cr(III)-bearing ferrihydrite is the expected product of Cr(VI) reduction by Fe^2+^_(aq)_ under circumneutral conditions [11, 48, 52]; however, as differences in Fe^2+^_(aq)_ concentrations are minimal (Table 1), this likely has little effect on the speciation of Cr in the product. A portion of the green rust is also expected to dissolve when added to the chromate solutions due to the relatively high solubility of green rust, which likely affects the reactions performed in this study. However, as the initial pH values of the chromate solutions are identical, meaningful differences in the behavior of each batch reaction due to green rust dissolution are not expected, but possible. Cr speciation is correlated to the fraction of Cr(III) oxidizible by δ-MnO_2_ after 14 days (Fig. 8b), but it is clear that oxMg-GR is an exception to the overall trend, as the fraction oxidized by δ-MnO_2_ is lower than predicted. Therefore, it is likely that the low Cr lability in this product is the result of other factors such as partial conversion to feroxyhyte instead of depending purely on the Cr speciation.

**Table 4:** Summary of Cr-XANES LCF fitting parameters

Weighing factors	Cr (OH)_3_	Cr-ferrihydrite	Sum	R-value	Reduced Chi squared
GR 7d	0.62 (4)	0.40 (4)	1.02	0.003225	0.00202
AlGR 7d	0.57 (5)	0.45 (5)	1.02	0.00584	0.00377
MgGR 7d	0.54 (5)	0.45 (5)	0.99	0.00693	0.00418
ZnGR 7d	0.66 (3)	0.36 (3)	1.02	0.00183	0.00116

**Fig. 9:**
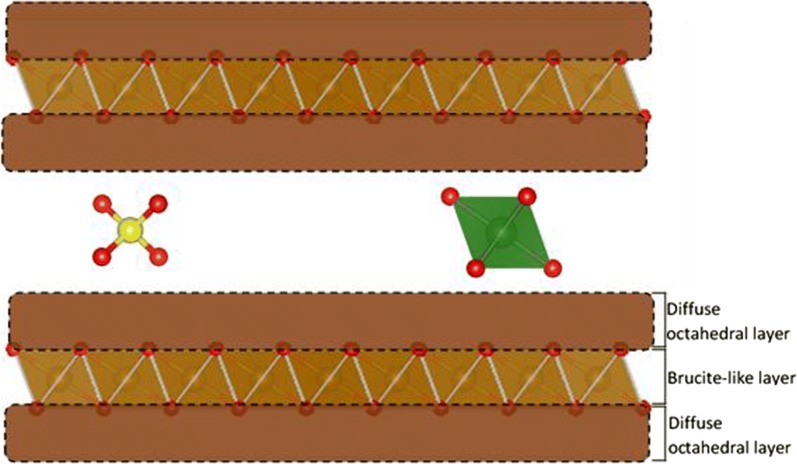
Probable structure of the reaction product. The brucite-like layers present in green rust are preserved, but some octahedra are displaced into the diffuse octahedral layer. The interlayer spacing is determined by the presence of sulfate, water and hydrated Cr (III)

Based on the XRD, PDF and XAS results, all oxidized samples appear to have maintained a layered structure composed of stacked SSI (Fig. 9) similar to those characterized by Yin et al. [56], with variable coherency and partial transformation to feroxyhyte in the case of oxMg-GR. Following oxidation (regardless of mechanism), the resulting strain causes displacement of Fe(III) octahedra into an interlayer region labelled in Fig. 9 as the diffuse octahedral layer. Fe octahedral positions within this layer are not defined in a unit cell, as Fe displacements are random, but the uniform geometry of Fe octahedra ensures that the diffuse octahedral layer has a uniform thickness, allowing the particle to maintain its periodicity in the [001] direction. The actual basal plane spacings depend on the species present in the interlayer, including water. In the presence of sulfate, the original structure remains, as the (001) reflection is still visible in a diffraction pattern of the undried samples (Additional file 1: Figure S1). However, displacement of Fe octahedra disrupts the hydrogen bonding in the basal layer that maintains the crystallographic coherence across layers, and as a result, many non-basal plane spacings are no longer detected or very faint in the diffraction patterns. These peaks in the XRD are broad, indicating poor coherence within the ab plane of the reaction products. In this case, linkage of adjacent layers and transformation to feroxyhyte is sterically inhibited by sulfate and the structure is better described as a metastable ferric green rust. However, if sulfate is removed by exchange for chromate prior to the Fe(II)–Cr(VI) electron transfer, adjacent layers can be linked by either covalent bonding with Cr(III) or hydrogen bonding with residual H_2_O; this can lead to the topotactic formation of other Fe oxides such as feroxyhyte. Feroxyhyte forms due to the linkage of adjacent layers, as the two phases have similar layered structures and hexagonal symmetries, so following linkage of two oxidized octahedral layers, only a slight reorganization is necessary for this transformation. This reaction mechanism was also proposed by Skovbjerg et al. [14] and our previous study [28], but appears to vary slightly depending on the initial chromium concentration, as higher concentrations favor chromate exchange for interlayer sulfate. The effects of initial chromium concentration on the substituted green rusts are unknown. In addition, the XRD patterns of some samples (oxMg-GR and oxGR, to a lesser extent) with higher non-basal plane crystallinity appear to have partially transformed to feroxyhyte, as characteristic feroxyhyte XRD peaks are visible in these diffraction patterns, although oxMg-GR is the only product in which this transformation is clear.

Cr(III) hydroxides are also present in the samples and may make a contribution to the PDF pattern between 0 and 10 Å, especially the correlations at 1.98 and 3.0 Å. A PDF pattern of Cr(III) hydroxide is not available; for peak positions and amplitudes, see Tang et al. [57]. Chromium reduced in the interlayer can form Cr(III) hydroxide domains ([Cr^3+^] is very high in the interlayer following reduction) or bind to adjacent diffuse octahedral layers. Interior Cr(III) hydroxide domains may have formed in oxMg-GR, as the low lability of Cr in this sample despite its relatively high Cr(III) hydroxide content suggests that this phase may be somehow sequestered. It should be noted that this is a metastable, transitional structure that will likely eventually transform to a mixture of Cr(III)-bearing goethite and Cr(III) hydroxide.

### Effects of cation substitution on reaction mechanisms and byproducts

**Fig. 10:**
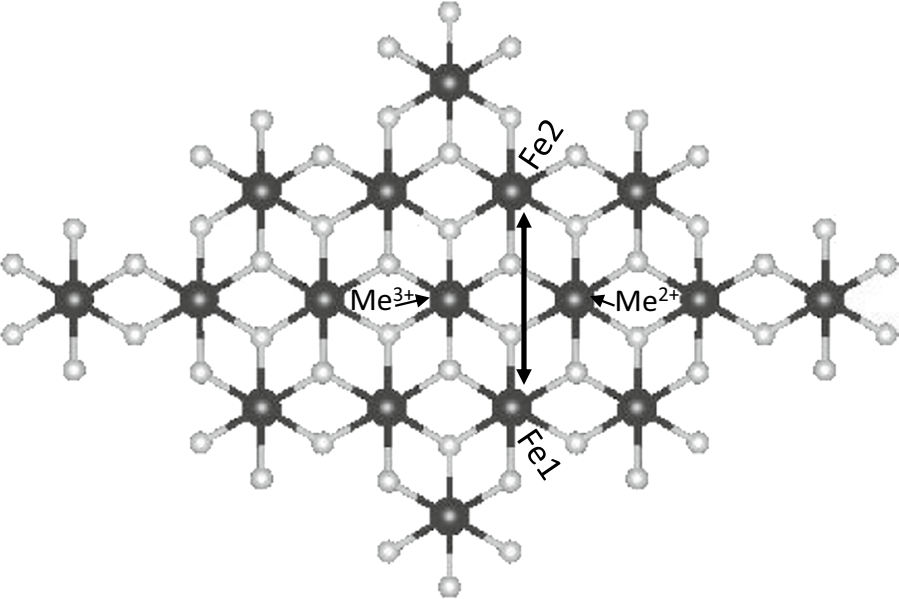
Brucite-like sheet used to demonstrate electron transfer between Fe-centered octahedra. Electron transport is between next-nearest neighbor Fe sites labelled Fe1 and Fe2, which are coupled via superexchange with atoms at the neighboring sites Me^2+^ and Me^3+^

Substitution of Al, Mg and Zn for Fe in green rust sulfate alters the morphology, structure and chemical properties of the mineral [36, 37, 59, 60], which could result in substantial changes in reactivity and reaction mechanism when exposed to hexavalent chromium. Green rust [29], like magnetite and other Fe oxides [33], is a semiconductor, potentially allowing reduction of chromate at its surface and rims by electron transport from within the particle”s interior. Conduction within green rust is best modelled by a “polaron hopping” mechanism that transports electrons and electron holes in a series of of Fe(II)–Fe(III) charge transfer steps rather than through a delocalized conduction band [29]. Density functional theory (DFT) modelling of polaron hopping within a green rust-like Fe(OH)_2_ plane showed that the potential rate of electron hole propagation at 300 K reaches 10^10^ s^−1^ in the case of transfer between next-nearest neighbor FeO_6_ octahedra (sites Fe1 and Fe2 in Fig. 10), 10^8^ times the rate of any other transfer mechanism. The most important parameter controlling the rate of charge transfer was the electronic coupling matrix element (V_ab_), which represents superexchange processes that link the electron spin states of magnetic cations (i.e. transmission metal cations with a net spin) covalently bound to a shared ligand [35, 61, 62] as a consequence of the Pauli exclusion principle. This coupling mechanism allows Fe^3+^ ligand field transitions, which drive electron transfer and would otherwise be forbidden by the spin-selection rule [33]. Next-nearest neighbor Fe atoms in green rust are not coupled directly by superexchange since they do not share a bridging –OH ligand, but are effectively coupled since both Fe octahedra participate in superexchange interactions with intermediate Fe(III) and Fe(II) octahedra (at sites Me^3+^ and Me^2+^ in Fig. 10). This rapid charge-hopping mechanism allows the regeneration of electron holes at the crystal edges resulting from chromate reduction and therefore continued reduction at the surface as long as it is not passivated. Oxidation by this mechanism would preserve the morphology and structure of the green rust, as it doesn’t require exchange of chromate for sulfate. The continued presence of sulfate in the interlayer sterically inhibits linkages between adjacent layers but maintains the particle structure by linking adjacent layers via electrostatic interactions. Antony et al. [63] observed a reaction product with this structure when oxidizing green rust sulfate with O_2_, and our previous study [28] found a similar result. On the other hand, if chromate exchanges for sulfate and is reduced by nearby Fe(II), a new 3-dimensional structure can form as adjacent layers are linked. In this case, transformation to other Fe oxides such as feroxyhyte (as seen in the present study) or goethite [14] is possible.

Cation substitution is expected to alter this intrasheet conductivity, depending on the properties of the substituted cation. Since Al^3+^, Mg^2+^ and Zn^2+^ are not capable of donating or accepting electrons, as they do not have additional stable oxidation states, incorporation of these cations along the electron transfer chain at sufficient concentrations (≥ 10%) can lower the rate of electron conduction to the particle surface [34, 64], and this effect may be stronger when electron transport is only possible in two dimensions. In addition, since these ions also have full or empty valence orbitals and therefore no net spin, they are unable to induce superexchange interactions with adjacent FeO_6_ octahedra when substituted at the Me^2+^ and Me^3+^ sites in Fig. 10 and therefore lower the rate of Fe(II)→Fe(III) charge transfer. Mg substitution appears to have this effect, as the partial transformation to feroxyhyte, lower levels of Cr(III) hydroxide in the Cr K-edge XAS spectra and low S concentrations measured in oxMg-GR by EDX suggest that chromate exchange for sulfate is taking place during these reactions, which leads to the formation of a more stable product. Al-GR also produces a product more resistant to oxidation by δ-MnO_2_ than pure green rust with a lower proportion of Cr(III) hydroxide. On the other hand, Zn substitution appears to have the opposite effect, as oxZn-GR was primarily composed of Cr(III) hydroxide and a layered, incoherently-stacked product as expected when Cr(VI) is reduced by electrons transferred to the crystal edges from its interior. As a non-magnetic cation, Zn^2+^ is also unable to couple the electron spin states of adjacent Fe-centered octahedra, and Zn substitution is expected to favor interlayer reduction of Cr(VI), and some evidence for this reaction exists in the form of the rim-like domains observed in the oxZn-GR TEM images. However, as Cr hydroxide is still the dominant Cr carrier phase in this sample, it is possible that other factors favor oxidative transformation of Zn-GR to a stacked SSI reaction product instead.

Additionally, cation substitution can lead to thermodynamic constraints on the reaction mechanism. For example, Zn^2+^ has a similar ionic radius to Fe^2+^ and is therefore easily incorporated at Fe(II) sites in green rust, phyllosilicates and other mixed-valence Fe oxides such as magnetite [65]. However, divalent metal substitution in Fe(III) (oxyhydr)oxides is generally not favorable because of its effects on the crystal field stabilization energy [66], differences in atomic radius, and effects on charge balance. Gerth [67] synthesized goethite with relatively high levels of Zn (Zn/Zn + Fe = 0.07), and Manceau et al. [68] identified natural goethite with approximately 2% substitution of Zn for Fe, so Zn incorporation into a Fe (III) oxide product is possible but most likely not thermodynamically favorable in this system, particularly because Zn substitution at the Fe(II) site is so high (13.5%). Instead, when ZnGR is oxidized by Cr(VI), the presence of a divalent cation with a higher ionic radius such as Zn^2+^ within the octahedral sheet may reduce the structural strain resulting from oxidation relative to other green rusts, possible including pure green rust sulfate. Therefore, the stacked SSI product is most likely more stable and crystalline, so chromate exchange for sulfate and collapse of the stacked SSI layers is less favorable. As a result, Cr(VI) reduction at the particle surface coupled to electron transfer from the interior is probably a more dominant reaction mechanism, producing Cr(III) hydroxide as the dominant Cr carrier phase in oxZn-GR.

## Conclusion

Laboratory-scale experiments are only the first step in developing and implementing an effective in situ remediation method, but the results presented here suggest that the controlled substitution of cations into green rust can significantly improve its ability to remediate hexavalent chromium contamination. In particular, the increased stability of the reaction products in the case of Mg substitution represents a significant improvement compared to existing particle-based in situ chemical reduction methods, and the reactants can be simply and inexpensively synthesized from sulfate salts available in bulk as agricultural chemicals. Mg^2+^ is abundant in soils and non-toxic, and due to its similar ionic radius to Fe^2+^ (0.072 nm vs. 0.078 nm, Shannon [69]), can easily substitute for structural Fe (II) and is a common substituent in green rusts identified in natural soils [37].

This technique is best applied by adding a large excess of ex situ-synthesized Mg-substituted green rust to a Cr-contaminated site. Excess Fe (III) allows the formation of more-crystalline Fe (III) oxides with lower levels of Cr incorporation [14], and excess Fe (II) can catalyze more rapid formation of these oxides [70–72] via a dissolution-precipitation mechanism. These oxides may also be a sink for other metal contaminants often associated with chromium contamination at former metal plating sites such as Ni^2+^, Cd^2+^ and Cu^2+^ [3]. In addition, under the reaction conditions tested in the present study (pH = 7.0), the green rust surface is positively charged [73], which should favor sorption of chromate at the particle surface followed by reduction. Synthesizing green rust under more alkaline conditions (pH > 8.0) may favor exchange of chromate for interlayer sulfate, as observed when reacting chromate with Fe (II)-bearing smectites [74, 75]. A study of these reactions at different initial chromium concentrations would also be useful for determining the reproducibility of these results under variable conditions. Finally, higher or lower levels of isomorphic substitution may also have an effect on the reactivities and reaction byproducts of these green rusts.

## Supplementary information

**Additional file 1.** Includes x-ray diffraction patterns obtained using benchtop equipment as well as a more detailed description of the XAS data analysis procedure.

## Data Availability

Data sets are available without restriction from the authors on request (andrew.thomas@kit.edu).

## Abbreviations

ISCR: In situ chemical reduction
TEM: Transmission electron microscopy
XAS: X-ray absorption spectroscopy
XRD: X-ray diffraction
PDF: Pair distribution function
ICP-OES: Inductively-coupled plasma optical emission spectroscopy
FWHM: Full width half maximum
XANES: X-ray absorption near-edge spectroscopy
EXAFS: Extended X-ray absorption fine-structure spectroscopy
SEM: Scanning electron microscopy
EDX: Energy dispersive X-ray spectroscopy
SAED: Selected-area electron diffraction
STEM: Scanning transmission electron microscopy
DFT: Density functional theory
GR: Green rust
SSI: Single-sheet iron oxided
