# Supplementary material for: Effects of metal cation substitution on hexavalent chromium reduction by green rust
Source: Geochem Trans. 2020 Feb 14;21:2. doi: 10.1186/s12932-020-00066-8 (PMC7020553; doi:10.1186/s12932-020-00066-8)
Supplement: Supplementary file 1 — Additional file 1. Includes x-ray diffraction patterns obtained using benchtop equipment as well as a more detailed description of the XAS data analysis procedure. [file 12932_2020_66_MOESM1_ESM.docx]

Effects of Metal (loid) Cation Substitution on Hexavalent Chromium Reduction by Green Rust

Andrew N. Thomas^1,*^, Elisabeth Eiche^1^, Jörg Göttlicher^2^, Ralph Steininger^2^, Liane G. Benning^3,4^, Helen M. Freeman^3,+^, Dominique J. Tobler^5^, Marco Mangayayam^5^, Knud Dideriksen^5^, Thomas Neumann^6^

^1^ Institute of Applied Geosciences, Karlsruhe Institute of Technology, 76137 Karlsruhe, Germany

^2^ Institute for Photon Science and Synchrotron Radiation, Karlsruhe Institute of Technology, 76344 Eggenstein-Leopoldshafen, Germany

^3^ GFZ German Research Center for Geosciences, Telegrafenberg, 14473 Potsdam, Germany

^4^ Department of Earth Sciences, Free University of Berlin, 12249 Berlin, Germany

^5^ Nano-Science Center, Department of Chemistry, University of Copenhagen, 2100 Copenhagen, Denmark

^6^ Department of Applied Geosciences, Technical University of Berlin, 10587 Berlin, Germany

^+^ Current Address: School of Chemical and Processing Engineering, University of Leeds, Leeds LS29JT, UK

**Appendix 1: Benchtop XRD characterization of reaction byproducts**

After terminating the reaction by filtration, samples were resuspended in acetone, and the suspension was transferred dropwise to a low-background Si sample holder before addition of glycerol to protect the sample from oxidation. Measurements were then performed using a Bruker D8 diffractometer. X-rays were emitted from a Cu-Kα source (λ = 1.5418 Å), and data were collected at 2θ values between 2-82° with a step size of 0.02° and an average counting time of 1 second per step. Background diffraction patterns were collected by measuring an empty sample holder, and the XRD-BS software was used to remove the background from the sample data.


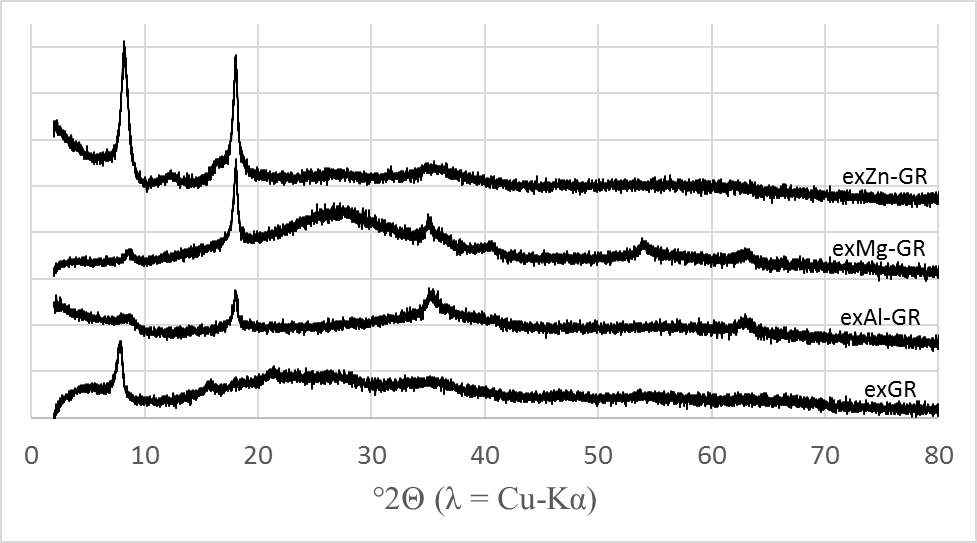


**Figure S1** X-ray diffraction patterns of reaction byproducts in Bragg-Bentano geometry.

**Appendix 2: EXAFS fitting strategy**

When fitting the Fourier-transformed Fe EXAFS spectra, the fitting procedure for feroxyhyte outlined in (1) was used due to the close similarity of these spectra to synthetic feroxyhyte. All data processing and fitting steps were performed using the Athena and Artemis software, both of which are part of the Demeter software package (2). First, all collected spectra were aligned by setting the energy of the first inflection point of a simultaneously-collected Fe metal foil reference spectrum to 7112 eV. After checking for significant differences resulting from inhomogeneity or beam damage, the three replicate scans collected from each analyzed sample were merged, and the merged file was used for all subsequent analysis steps. The merged file was then normalized by calculating third order pre- and post-edge background functions, subtracting this background, and setting the edge jump of the spectra to one to allow comparison of the resulting norm(E) spectra. This allows representation of the spectra in χ (*k*) space, which shows the deviations of the post-edge spectrum from the background in terms of *k*, the photoelectron wavenumber. When displaying the spectra in χ (*k*) space, differences in the spectrum quality became obvious; a Co K-edge is visible in the exGR spectrum, and several other spectra are noisy above *k*=12. For this reason, the Fourier transformation boundaries vary between samples so that these features do not harm the Fourier-transformed spectra (i.e. R-space).

The fits of the Fourier-transformed spectra were performed using phase and amplitude functions calculated from a hematite theoretical structure (3), similar to the procedure from (1). The spectra were fit progressively, one shell at a time. The first coordination shell, which corresponds to a Fe-O scattering distance of 1.98 Å, was fit by fixing the coordination number at 6, corresponding to the accepted structure of feroxyhyte (1,4,5). This fit yielded acceptable values for the amplitude reduction factor (S_0_^2^), the half path-length and the Debye-Waller factor (σ^2^) (see Table 3 in the main text). The second, broad shell is composed of two scattering paths at approximately 3.04 and 3.4 Å, corresponding to the Fe-Fe corner- and edge-sharing geometries in goethite. However, constructing an acceptable fit was only possible when the values of certain parameters were constrained. When fitting the first Fe-O shell at 1.98 Å, the fitted S_0_^2^ value converged at approximately 0.9 for all samples; therefore, this parameter was fixed at 0.9 to allow determination of the coordination numbers in the second shell. Similarly, although there is some disagreement concerning the placement of Fe within feroxyhyte’s structure, more recent studies (1,5,6) agree that only octahedrally-coordinated Fe is found within the feroxyhyte structure. Therefore, the coordination number of the first shell was fixed at six. Finally, initial attempts at fitting these spectra yielded unphysical results due to correlations between the coordination numbers and Debye-Waller factors in the second shell. To solve this problem, both scattering paths were constrained to share a σ^2^ value of 0.015, similar to fits performed on ferrihydrite spectra collected under identical conditions.

**References**

1. Manceau A, Drits VA. Local Structure of Ferrihydrite and Feroxyhyte by EXAFS Spectroscopy. Clay Miner. 1993 Jun 9;28(02):165–84.

2. Ravel B, Newville M. ATHENA and ARTEMIS Interactive Graphical Data Analysis Using IFEFFIT. Phys Scr. 2005;T115:1007–10.

3. Blake RL, Hessevick RE, Zoltai T, Finger LW. Refinement of the hematite structure. Am Mineral. 1966 Feb 1;51(1–2):123–9.

4. Sestu M, Carta D, Casula MF, Corrias A, Navarra G. Novel interpretation of the mean structure of feroxyhyte. J Solid State Chem [Internet]. 2015;225:256–60. Available from: http://dx.doi.org/10.1016/j.jssc.2015.01.003

5. Patrat G, de Bergevin F, Pernet M, Joubert JC. Structure locale de δ-FeOOH. Acta Crystallogr Sect B Struct Sci. 1983 Apr 1;39(2):165–70.

6. Pernet M, Obradors X, Fontcuberta J, Joubert J, Tejada J. Magnetic structure and supermagnetic properties of &amp;#948;-FeOOH. IEEE Trans Magn. 1984 Sep;20(5):1524–6.
